# Conceptualizing Care Continua: Lessons from HIV, Hepatitis C Virus, Tuberculosis and Implications for the Development of Improved Care and Prevention Continua

**DOI:** 10.3389/fpubh.2016.00296

**Published:** 2017-01-10

**Authors:** David C. Perlman, Ashly E. Jordan, Denis Nash

**Affiliations:** ^1^Icahn School of Medicine at Mount Sinai, Mount Sinai Beth Israel, New York, NY, USA; ^2^Center for Drug Use and HIV Research, New York, NY, USA; ^3^Department of Epidemiology, School of Public Health, City University of New York, New York, NY, USA; ^4^Center for Drug Use and HIV Research, New York, NY, USA

**Keywords:** care continuum, cascade of care, HIV, hepatitis C, treatment and prevention

## Abstract

**Background:**

To examine the application of continuum models to tuberculosis, HIV, and other conditions; to theorize the concept of continua; and to learn lessons that could inform the development of improved care and prevention continua as public health metrics.

**Methods:**

An analytic review of literature drawn from several fields of health care.

**Results:**

The continuum construct is now part of public health evaluation systems for HIV, and is increasingly used in public health and the medical literature. Issues with the comparability and optimal design of care continuum models have been raised, and their methodologic and theoretic underpinnings and scope of focus have been under-addressed. Review of relevant publications suggests that a key limitation of current models is their lack of measures reflecting incidence and mortality. Issues relating to continua data being longitudinal or cross-sectional, definition of numerators and denominators for each step, data sources, measures of timeliness of step completion, theoretic models to facilitate inferences of causes of care continuum gaps, how measures of prevention efforts, reinfection/relapses, and interactions of continua for co-occurring comorbidities should be reflected, and how analyses of differences in retention over time, across geographic regions, and in response to interventions should be conducted are critical to the development of sound care and prevention continuum models.

**Conclusion:**

Lessons learned from the application of continuum models to HIV and other conditions suggest that the application of well-formulated constructs of care and prevention continua, that depict, in well defined, standardized steps, incidence and mortality, along with degrees of and time to screening, engagement in care and prevention, treatment and treatment outcomes, including relapse or reinfection, may be vital tools in evaluating intervention and program outcomes, and in improving population health and population health metrics for a wide range conditions.

## Background

The construct of “care continua” has become an important tool in the evaluation and improvement of the overall care for certain conditions (1–5). Examinations of prevention and care continuum constructs have provided valuable insights into the progress of individuals and populations through sequential steps of care, and into barriers to such progress. While the construct has been in use for some time, it has received increased attention and formalized acceptance as part of the US’ “HIV care continuum initiative” (3) and has now been integrated as part of formal public health evaluation systems for HIV. The construct is also now a central component of the Joint United Nations Programme on HIV/AIDS (UNAIDS), and World Health Organization approaches to HIV global public health; the UNAIDS goal of 90% diagnosed, 90% on antiretroviral therapy (ART), and 90% virologically suppressed (“90–90–90”) implicitly adopts a care-continuum framework (6). In fact, the HIV continuum model has been referred to as an effective and important tool “for improving the health of people living with HIV and for achieving the public health benefits of ART” (7) and for “measuring the performance of HIV care and treatment programs (8).”

The construct of care continua is also being increasingly used in other clinical and public health settings, such as in evaluations of care systems for other infections, such as hepatitis C virus (HCV), sexually transmitted infections (STIs) (5, 9, 10), and non-communicable diseases (e.g., diabetes) (11, 12). However, issues with their comparability and optimal design have been raised, and their methodologic and theoretic underpinnings and their scope of focus have been under-addressed (1, 13, 14). Further, despite their increased use, continuum constructs have not been formally or consistently applied to a wide range of other conditions that might benefit from their use. This includes some conditions that may not themselves currently be viewed through a continuum of prevention and care lens, but that are frequently identified as barriers to progress through the steps of continua of other conditions, such as evaluations identifying an adverse impact of substance use, misuse, and substance use disorders (SUDs) on the HIV or HCV care continua (5, 15, 16).

Our goal is to more fully theorize the continuum construct and to help develop understandings, definitions, and applications of care continua so as to improve their use as valuable tools for scientific, programmatic and public health evaluations and interventions generally. To achieve this goal, we will examine some examples of the valuable application of continuum models and explore issues with and limitations of existing models and make suggestion for their improved use.

## Methods

Examined literature comes from several fields of social science, health care and public health over several decades. In order to explore the continuum construct through a variety of lenses, we adopted an analytic and synthetic approach to draw lessons from these diverse sources. This approach relied on traditional literature review search methods and on the application of the case study methodology and the comparative method (17, 18). These methods have been used extensively in social and political science (19, 20). They rely on comparisons between and inferences drawn from a modest number of cases (in this analysis; “cases” refers to publications or applications of continuum constructs) (20). We then sought to review the historic development, construction, and application of continuum models, the delineation of specific steps, observations about the clinical and public health domains represented and not represented in the models, and definitions and data sources.

## Results

### Early Applications and Basic Considerations

The concept of care continua derive from the Piot and Piot-Fransen models for tuberculosis (TB) and STIs, respectively, where models focused on operational considerations that arose and that reduced the overall effectiveness of clinical and public health efforts already demonstrated by studies to be efficacious in idealized settings (4, 21, 22). The Piot-Fransen model has been used to understand systems of STI care and the potential impact of various interventions by considering, for example, a population of women and the proportion (1) with an STD, (2) who are symptomatic, (3) seek treatment, (4) go to a health unit, (5) are treated correctly, (6) are adherent, (7) are treated effectively, and (8) whose partners are treated (21).

Continuum models are usually graphically represented as a bar graph where each bar represents the proportion of persons completing each step (3, 9, 23–25). In such depictions, if the numerator in the first step is then taken to be the denominator of the subsequent step (9), each step is essentially represented as a separate event and the impact of cumulative losses through sequential steps is visually de-emphasized. If, instead, the denominator is kept constant throughout sequential steps, the overall impact of cumulative losses is made visually more apparent (25). Hayes et al. depicted the progressively decreasing proportions of persons completing each step, using visual descriptions of quantitative data to assess not only the losses occurring at each step but also to allow estimates of the impact of different strategies, such as the addition of interventions including active case finding, syndromic treatment, or mass community-level treatment, on continuum progress and the impact of such interventions on population health (21).

### Valuable Applications of Continua Constructs

HIV likely represents the most established and successful application of a continuum model. More recent applications of continuum models for the prevention of maternal-to-child transmission of HIV and for HIV care generally (26, 27) yielded valuable insights and introduced methods for both measuring the progress of individuals and populations (2, 3, 15, 23). The Centers for Disease Control and Prevention (CDC) formally uses two well-defined approaches to evaluate the HIV continuum models with the specific intent of both gauging progress toward specified public health goals and of directing HIV prevention resources most effectively (3). Both continuum models consist of the same five steps but employ different denominators. The steps specified in both of these CDC models are the proportions diagnosed, linked to care, engaged or retained in care, prescribed antiretroviral therapy, and virally suppressed (1, 3, 28). These steps correspond to measurable outcomes with clinical and public health relevance.

One model, “the prevalence-based HIV care continuum,” examines each specified step as a proportion of the total number living with HIV including those diagnosed and those undiagnosed. The other model, “the diagnosis-based HIV care continuum,” uses as a denominator the total number of those diagnosed with HIV excluding those who have not been diagnosed. The prevalence-based model can be used to assess outcomes for broad populations such as young women, but not subgroups of these populations, e.g., low-income young women. In contrast, the diagnosis-based model allows examination of more detailed population subgroups referred to as stratified continuum models.

Similarly, continuum models are also proving valuable in identifying gaps and focusing resources for HCV, TB, and other services (5, 11, 29). Analyses of the TB continuum led to the observation that non-adherence to TB treatment was one key obstacle to effective completion of TB treatment (30); directly observed therapy for TB was developed in response to this observation to address treatment adherence, to promote progress through the TB continuum and reduce the development of acquired resistance, and to reduce ongoing transmission (31, 32). Indeed, continua conceptual models for HIV and HCV, and strategies to promote engagement and retention, and deter the development of acquired drug resistance, have been potently informed by global experience with, and continuum-based analyses of, TB public health systems and the strategies employed.

Despite the contributions already made through the use of continuum models in HIV and other fields, there are a number of key issues central to their optimal use as evaluation tools generally that require further consideration. These include issues of theory; the delineation of specific steps standardization; reflecting incidence, time, and disease-specific morbidity and mortality; and statistical analysis of the models that will be addressed in the sections that follow.

### Use of Theoretic Frameworks

An identification of gaps does not in and of itself provide an understanding of the reasons for such gaps. An understanding of reasons for gaps in or barriers to the progress of individuals or populations through the steps of care requires the application of an appropriate theoretic framework. Where continuum analyses are guided by an appropriate theoretic model, factors affecting progress through sequential steps can be more fully examined; for example, some studies demonstrate the importance of structural- and individual-level factors as determinants of progress through continua (16, 33). This is important because the framing as “care” continua focuses attention primarily on clinical or biologic endpoints and may, therefore, de-emphasize key explanatory factors that fall outside of traditional medical care systems. Eco-social theory and the population health approaches of Krieger and Rose, respectively, may be valuable underlying theoretic models (34, 35). This may facilitate the identification of multi-level factors impacting individual progress through the continuum, as well as examine how progress through continuum steps reflects and impacts population-level health (36).

There are frequently large time gaps between demonstrations of efficacy and implementation in practice (37). Continua models can help focus attention on issues critical to the effectiveness and implementation of care and prevention interventions (31). For example, there are highly efficacious drugs available to treat TB (i.e., they work very well under optimized conditions). However, despite this, there are many impediments to TB elimination which are predominately “operational,” i.e., the real-world effectiveness of these efficacious drugs is reduced by a range of multi-level barriers (38, 39). Similarly, there are also now highly efficacious drugs for HCV offering the potential for a cure; however, here, the real world effectiveness of these agents is undercut by issues of cost and resultant processes that restrict access (40, 41). Continuum models viewed through appropriate multi-level theory are useful in identifying and guiding efforts that address barriers and disparities.

### Defining the Steps in a Continuum Model

The steps of any specific continuum model should reflect the specific clinical features of that condition and actual processes of prevention and care and should be chosen to facilitate elucidation of potential barriers to progress through the continuum so that discrete barriers can be addressed. Many continua models begin with an initial step of awareness of risk or a condition and whether individuals may seek attention or of active testing, case finding, or screening of either high-risk or general populations to identify those with the condition in question (5, 42). Commonly these steps need to be followed by steps of further evaluation and engagement and retention in care. The construct “seek, test, treat, and retain” is one HIV continuum paradigm that emphasizes this common sequence of steps (43). However, in these constructs, the category “test” or “screen” may mean a range of things that may need to be subdivided into components for optimal monitoring, implementation, and improvement. For example, testing can refer to HCV antibody testing with or without confirmatory viral load testing. Clear characterization of each step can allow specific decisions to be made about choice architecture questions, such as whether testing is offered as “opt in” or as “opt out” (the use of specific defaults can significantly improve specific care continuum steps) or whether confirmatory testing, where needed, should be done as an automatic reflex (44–46).

### Reflecting Incidence in Continua Models

One key limitation of beginning a continuum model with the steps of seeking or testing is that the model then may fail to reflect a key public health indicator of disease: incidence. Continuum models often begin with the proportion of a general or known-to-be-positive population who are screened (3, 28). Thus, screening does not distinguish between prevalent or incident cases. Further, models that start with the number of positive cases, or those diagnosed as the denominator (as in both CDC HIV models), rest entirely on prevalence. Implicit in standard HIV continuum models is the notion that increases in the proportion of individuals tested and treated will translate to reductions in incidence; however, the failure to distinguish between prevalent and incident cases and the failure to reflect measures of incidence in the model inappropriately de-emphasizes the importance of incidence as a public health indicator, may obscure detection and consequences of changes in incidence, and may contribute to suboptimal allocation of resources. Distinguishing between prevalent and incident cases and to reflect measures of incidence in continuum models would improve their value as public health tools.

### Reflecting Prevention in Continua Models

Continua constructs may be valuably applied to prevention as well as to care where initial testing or screening are crucial both in identifying those affected by the specified condition and those who may be at risk for but not yet have the condition (8). Most continuum models exclude those testing negative from subsequent consideration. Those who test negative for HIV, or other specified conditions, may, nonetheless, be at risk and consequently require linkage to prevention services, require retention in such services, and may require efforts at adherence support for risk reduction interventions. In fact, the reliance on a step of testing or screening in many continua models, which may identify both those already affected and those at risk, highlights the importance of understanding continua models as more than a linear progression of steps of those affected, but of seeing care and prevention as linked and inter-related. As McNairy and El-Sadr highlight, the step of testing and screening serve as a critical point of intersection between the HIV prevention and care continua models (8); however, developing separate prevention and care continuum models may miss or obscure the bi-directional relationships between prevention and care that might be revealed by an integrated model. Consequently, one key consideration for delineating steps in a continuum model is that of appropriately representing factors that relate to primary prevention.

### Connections between Diagnosis and Treatment in Continua Models

The issue of the connection between screening and acting on the results of screening was highlighted by John Sbarbaro in an editorial entitled “To seek, find, and yet fail” written in response to a novel TB skin testing program, which identified a high prevalence of latently infected persons and yet included no efforts to link such persons to evaluations to exclude active TB or initiate treatment of latent TB infection (47). This issue of ensuring effective connections between steps of identification and diagnosis, and subsequent linkage to and initiation of care, emerge as common and critical to care continua for multiple conditions. Approaches to reducing gaps after screening include interventions, such as patient navigators, or of locating testing and care in community settings or in settings where specific risk groups convene (48). Significant experience in HIV, HCV, and TB care suggests that processes of passive referral to treatment after screening yield inferior linkage outcomes compared with systems of active linkage (10, 49, 50).

Further, diagnostic evaluations are often individualized by providers influenced by hidden cognitive processes related to the providers’ estimation of a patient’s resources, often within constraints imposed by patients, organizations, and insurers (51). These organizational constraints (e.g., prior authorizations) may themselves pose barriers to medical evaluation completion and care continuum progress (40, 52, 53). Similarly, providers, in response to guidelines or unconscious biases, differentially apply “eligibility criteria” in ways that constituting a “stutter-step” in health-care provision, and introducing health disparities (25, 51). Further, these evolving factors may lead to variability that poses issues for standardized analyses.

### Methodologic Issues in Continuum Models

Lourenco et al. have noted that HIV continuum models used different countries vary both in enumerated steps and definitions of steps and argue for the need for standardization of the HIV continuum construct to allow continuum comparisons (14). As discussed in Section “Early Applications and Basic Considerations,” the clearly delineated and well-standardized two CDC HIV continuum models have proven to be valuable constructs. Yet, while these two main models may provide different and complementary information, since the numbers of persons with HIV who have not been diagnosed can only be estimated not directly measured, they may give different estimates for proportions at each of the defined steps (54). Further, both models are based on cross-sectional surveillance data and evaluate outcomes for the aggregate population studied in a single year and do not necessarily reflect individual-level continuum progress. Another model being applied in some settings is to examine the continuum among those with newly diagnosed HIV (another potentially valuable stratification); this model facilitates incorporating considerations of time elapsed between steps, such as the proportion achieving viral suppression in the 12 months following diagnosis.

A recent systematic review of publications examining HIV continuum models focusing on data sources, methodology, and study comparability with respect to these parameters (13). Analyses were restricted to published data of HIV care continua in well-defined populations, and further restricted to studies providing viral suppression data and explicit methods. Despite these rigorous inclusion criteria, the authors identified significant variability both in the number of steps delineated, and in the data source used for steps reported on in the included studies. There was also moderate variability in the definitions used to delineate both numerators and denominators at each of the steps (e.g., different approaches were taken to handling persons who may have died, moved away, or been incorrectly notified). A variety of time frames or attendance requirements were used in defining linkage to care, and there was heterogeneity in definitions of what contributed to the numerator of those “on ART,” as well as in what cut-off constituted virologic suppression. The authors point out the potential value of comparisons of continuum analyses stratified by geographic regions and time, and among groups, but stress the critical importance for consistency in methodology and definitions to make such comparisons valid.

Another recent paper examined an 8-step HIV continuum model and conducted sensitivity analyses based on variations in continua definitions (55). They found that requiring or not requiring CD4 cell count and viral load testing as part of definitions of being linked to care changed proportions of those doing so up to 18%. Definitions of being on ART (which required at least two medication dispensations at least 3 months apart) yielded rates 20% lower than definitions requiring any ART in that calendar year. Overall, they found that the step most sensitive to variations in definition was that of the last step, viral suppression. A rigorous definition requiring two suppressed viral load determinations at least 3 months apart classified 49% as virologically suppressed, a proportion 15–25% less than definitions requiring a single undetectable viral load.

Overall, these issues highlight the potential impact of variations in definitions on measured outcomes and the need for definitional clarity and sensitivity analyses.

### Representing Time in Continuum Constructs

In current continuum models, the depiction of sequential steps along an axis does not represent the time required for transitions from one step to the next. For example, the HIV continuum does not convey the fact that many people are diagnosed late in the course of their infection (52). It also does not convey the timeliness of linkages to care or of achieving viral suppression. Both of these are central to the success of treatment as prevention yet they are absent from typical HIV continuum constructs. Developing models that measure the time between screening, treatment, and treatment outcomes would be important.

Another key aspect of prevention and care relates both to the defined population and to the recognition that populations are not stable overtime (56). For example, continua models are often applied to specific geographic regions and individuals may move into or out of regions, with consequences both in interruptions in their own individual progress through continua and with implications for how to accurately count such individuals. These considerations may lead to inaccurate assessments of the proportions of persons at any given continuum step and require standardization to allow appropriate comparisons across geography and over time (13). With respect to HIV continua, this issue has been referred to as “churn” and has been recognized as leading to inaccurate estimates of the number of HIV-infected persons and hence, their representation in continua models (54, 57).

Another issue is whether continuum analyses examine cross-sectional or longitudinal data, a critical distinction where achieving outcomes takes variable amounts of time and where outcomes must be sustained overtime. Colasanti et al. examined continuous retention in care and continuous viral load suppression over 36 rather than 12- or 24-month periods (1) using generalized estimating equations with a logit link and Poisson regression log-link tests to evaluation retention in care and the prevalence ratio of viral suppression over time, respectively (58). Single cross-sectional analyses were found to potentially overestimate retention and virologic outcomes, and that longitudinal continuum constructs may better describe long-term outcomes and reveal disparities potentially obscured by cross-sectional annual examinations.

Use of continuum models that reflect longitudinal rather than cross-sectional data may be particularly important for understanding chronic conditions. For example, if a gonorrhea or syphilis case is diagnosed in any given year (where treatment can be a single dose or a brief course), treatment and cure should be obtained within that same year; a cross-section analysis would reflect this. Alternatively, for conditions requiring longer treatment (e.g., HIV), cross-sectional analyses may overestimate continuum progress.

### Endpoints for Continua Models: Relapse, Reinfection, and Mortality

For both HIV and HCV, a key continuum “endpoint” of viral suppression will remain relevant and vital. The rationale for the importance of this endpoint is the excellent data that viral load suppression translates both into individual-level quality health outcomes and population-level conditions that result in lower likelihoods of transmission with subsequent reductions in incidence which might then lead to epidemic control, elimination, and theoretically eradication (59, 60). Post-viral load suppression for HIV or HCV, it will be important to assess rates of relapse or reinfection. Similarly, for TB continua models, the common endpoint is a mixture of smear and culture conversion and completion of a duration of therapy shown in efficacy studies to translate to low rates of treatment failure, relapse, and secondary transmission. Yet while such valuable biological measures are generally depicted as continua endpoints, a critical appraisal of care continua suggests the need to reflect reinfection and relapse.

Further, virologic suppression or any other biologic or behavioral outcome measures are at best surrogates of the fundamentally more critical endpoint of mortality. The nineteenth century physician and epidemiologist William Farr noted that “the death rate is a fact. Everything else is an inference” (61). Issues such as whether antiretroviral treatment for HIV infection initiated promptly at diagnosis regardless of CD4 count and taken with adherence for decades translates to overall improved mortality, or whether competing increases in cardiovascular or other forms of mortality, associated or not associated with long-term ART use, may emerge, are open questions and would be missed by continuum analyses that stop at an outcome of viral suppression. Therefore, continua models would be more meaningful if the final step represented overall mortality or mortality attributable to the process being evaluated.

### Interacting Continua for Multiple Conditions

Another issue to be addressed relates to the reality that individuals may have more than one health condition, so that an individual may in fact be moving through multiple continua which may be interrelated to varying degrees. For example, for individuals with HIV/HCV, coinfection will be considered as part of both HIV and HCV continuum, respectively, and at an individual-level will need to move through the steps of both as part of optimal health care.

Movement through continuum for two or more conditions are likely to be impacted by the specifics of the service delivery systems, which would include whether care occurs through an integrated system that addresses all of the conditions or whether care is delivered through separate systems. Broadly speaking, this relates to the issues of vertical versus horizontal models of care and public health funding streams (62, 63). Specifically, it relates to a wide variety of models of co-located services such as whether HCV care is delivered within an HIV and SUD care setting or if individuals are referred elsewhere (64). In fact, use of continuum models for single conditions in some sense reproduces and reinforces systems of vertical care which have the potential to both create barriers themselves and to reframe and de-emphasize aspects of care for persons with commonly occurring comorbidities. To the extent that certain conditions assessed through the use of continua are highly overlapping, the use of a disease-specific continuum to inform resource allocation may be misleading or may miss opportunities for enhanced efficiencies (65).

Movement through one specific continuum may also be directly tied to movement through another continuum. Providers may make the decision to prioritize achieving HIV viral suppression prior to the initiation or consideration of HCV treatment; in fact, some treatment guidelines and insurance policies suggest or “mandate” this (40, 53). In that sense, achieving certain steps of HIV prevention and care might appropriately be construed as part of a HCV continuum model. Similarly, care systems for HCV may require screening for and treatment of SUDs (52). As an example of interacting care continua, for persons with HIV-related TB, the fact that TB treatment should precede ART initiation, and that in analyses the need for TB treatment is associated with late ART initiation, demonstrates that progress through these two care continua are linked (66). This highlights the need for models that clearly delineate the relationships between progress through co-occurring care continua.

Understanding that conditions identified as potential barriers to progress through a continuum for one condition may in fact be disorders requiring intervention, may allow improved understanding of the interaction of care systems and of how conditions may act as barriers, and may allow the development of more refined variables.

### Data Sources for and Analyses of Continua Constructs

Central to the development of any continuum model is the issue of identifying appropriate data sources for each of the identified steps. For HIV and TB, such data sources are reasonably well developed (67). Current models have relied on combinations of information derived from laboratory-based reporting and clinical care data, data which are often collected for specific health-care systems or regions, which directly inform continua constructs that describe these jurisdictions. Their use to populate models that combine jurisdictions may lead to inaccurate estimates unless careful methods are applied to identify duplicate cases (54). Large national databases and electronic medical record systems may be useful data sources for continua and further may facilitate the examination of interconnections between related models.

In formal analyses, continuum progress could be viewed a sequential ordinal variable where earlier stages are prerequisites for later stages (e.g., there is no HCV treatment initiation without HCV medical evaluation completion). Several formal quantitative analytic methods may be particularly valuable. In formal analyses, achieving sequential continuum steps may be viewed as a count variable. One approach could be to use the continuous ratio model (CRM) which is well suited to sequential outcomes of this type (68). With a logit link, exponentiated regression coefficients could indicate how the odds of progressing to a sequential continuums stage are affected by an intervention, in terms of an odds ratio. The CRM is equivalent to discrete-time survival analysis (69–71) where a continuum step would correspond to time periods in survival analysis. Further, for longitudinal continuum analyses, generalized estimating equations with a logit link or Poisson regression log-link tests, as employed by Colasanti et al. may be valuable (1, 58).

In analyzing progress through sequential steps is the consideration of whether each step requires comparable effort or results in comparable public health impact. Steps then may need to be weighed based on the varying on these considerations. One consideration is also whether the steps of a continuum should (or do) represent critical individual-level or population-level milestones, or whether they primarily reflect measurable milestones, and these may not be the same thing. It might be that shifting a continuum curve in which an improvement of some proportion at one step may not translate to relevant gains in population health, while an improvement of the same proportion at another step might do so (35). This may especially be so if the continuum is primarily focused on care and not on prevention or other public health metrics, such as incidence, timeliness of achieving specified outcomes, or deaths among those out of care. Hence, at an individual-level, weights might be assigned to steps on the basis of estimates of their relative degrees of difficulty, and at a population-level weights might be assigned on the basis of estimates of their relative importance to population health gains.

This leads to the issue of whether continuum steps are of necessity sequential, or should always be viewed so representationally or analytically. Qualitatively, it has been abundantly noted that individuals may complete any given step of a continuum model and for a range of reasons, not proceed to the next clinically logical or desired step, but then at some subsequent point in time become re-engaged at the same or even re-enter at an earlier step in the continuum model (72). Such stalled or backward movement may be the result of various barriers introduced by both providers and patients (e.g., losing health insurance) as part of a “stutter-step” (51). In most continuum models, the presumption is that those represented in the numerator of one step of necessity were in the numerator of the prior step. Yet, Magnal et al. identified individuals in an HIV continuum analysis who did in fact meet definitions for having been prescribed ART or even being virologically suppressed but did not meet definitions for having been retained in care (24). In their comparison, categorization of continuum stages as dependent subsets of prior stages led to underestimates of those prescribed ART or achieving viral suppression.

An underutilized potential of continuum analyses is their application to evaluating the impact of interventions to improve the steps of care. Hayes et al. used graphical representations of STI continuum outcomes to estimate the potential impact of different public health strategies on STI outcomes (21). Quantitative analyses might consider interventions as either time-invarying or time-varying exposures. In quantitative analysis, interventions attempting to improve progress through sequential continuum steps might in theory be viewed as a time-invarying exposure reflecting being on treatment or not. However, because intervention components may vary in content or emphasis through each step, interventions addressing multiple steps may be better viewed as a time-varying exposure. Similarly, covariates may have differential impact on different steps and, therefore, may represent time-varying confounders, while also potentially being on the causal pathway. This suggests that marginal structural models may be valuable analytic strategies for evaluating the impact of interventions (70, 73).

## Discussion

### Implications for Improved Use of Continuum Models

Review of the literature suggests several key implications for the improved use of continua models as clinical and public health tools. Models would optimally reflect incidence and distinguish incident and prevalent cases. They should reflect disease-specific and all-cause morbidity and mortality. Optimal models would reflect relapses and reinfections, as well as measures of primary and secondary prevention. Models should also reflect the timeliness between steps and have explicit and appropriate definitions, data sources, and means for handling those who move or die. Models should be understood to require the use of theoretic frameworks that consider structural as well as individual causes of identified gaps.

Numerous issues in the delivery of care and prevention for many conditions resemble those identified in the HIV, HCV, and TB continua, including issues of underdiagnoses, gaps in linkages between screening and initial diagnosis and engagement in treatment, issues in treatment retention, adherence, and relapse, and the interdigitation of continua for relevant comorbidities. Systems of care and prevention for many conditions can appropriately and usefully be viewed as consisting of a care and prevention continuum including steps of incidence, screening/identification, medical/psychosocial evaluation for treatment, engagement in evidence-based treatment, retention in treatment through to well-defined measures of treatment success, as well as degrees of engagement in evidence-based interventions to prevent relapse, and measures of overall and substance-related-specific mortality. It would be critical to define the denominator most relevant to each specified step. As with HIV and other continua, the use of various population denominators will be important in addressing different questions. It would be essential to identify appropriate data sources for each step, relevant and valid measures of treatment success, standardized definitions of numerators and denominators, and standard methods to account for those who move or die, and handling missing data. It would also be appropriate to reflect relationships between a given continuum and continua for key comorbidities (57, 74).

### Limitations

In reviewing the literature from several fields using traditional literature review search methods, some important contributions may have been missed and selection bias could have introduced. However, the review of literature from multiple fields serves as a form of triangulation which may ameliorate this risk (75, 76). Some topics meriting discussion may not have been optimally covered; decisions were made in an effort to balance comprehensiveness and focus. Further, the goals of and specific considerations relevant for models intended specifically for questions unique to either specific health-care systems or to unique populations within the substance use field may not have all been fully incorporated.

## Conclusion

Well-constructed and standardized continua models are proving to be invaluable for program development, evaluation and policy, for public and private health systems in standardizing and evaluating outcomes, informing study design, modeling, resource allocation, and for facilitating standardized comparisons of an expanding range of health outcomes across programs, states, and countries. Review of lessons learned from the valuable application of continuum constructs suggests that steps of the awareness, of screening, of linkage to evidence-based treatment and retention in such treatment, and of monitoring timely movement between steps, incidence, relapse/reinfection, and mortality. How best to reflect some of these factors will require more consideration and development. Identifying optimal data sources for continuum steps and standardizing definitions for these steps and of relevant numerators and denominators will be needed. Similarly, optimizing methods for quantitative analysis of progress through continua and of the impact of interventions on such progress is also needed.

In conclusion, the application of well-formulated constructs of care and prevention continua, that depict, in well defined, standardized steps, incidence and mortality, along with degrees of and time to screening, engagement in care and prevention, treatment, and treatment outcomes including relapse or reinfection, may be vital tools in evaluating intervention and program outcomes and in improving population health and population health metrics for a wide range conditions.

## Author Contributions

DP, AJ, and DN analyzed and interpreted the data. AJ wrote the first draft of the manuscript. DP, AJ, and DN read and contributed to multiple versions of the manuscript. All the authors read and approved the final manuscript.

## Conflict of Interest Statement

The authors declare that the research was conducted in the absence of any commercial or financial relationships that could be construed as a potential conflict of interest.
